# IFNγ Regulates Activated Vδ2+ T Cells through a Feedback Mechanism Mediated by Mesenchymal Stem Cells

**DOI:** 10.1371/journal.pone.0169362

**Published:** 2017-01-11

**Authors:** Karoline Fechter, Akaitz Dorronsoro, Emma Jakobsson, Izaskun Ferrin, Valérie Lang, Pilar Sepulveda, Daniel J. Pennington, César Trigueros

**Affiliations:** 1 Fundación Inbiomed, Foundation for Stem Cell Research, Mesenchymal Stem Cell Laboratory, Paseo Mikeletegi, San Sebastián, Spain; 2 Fundación para la Investigación Hospital Universitario La Fe, Valencia, Spain; 3 Blizard Institute, Barts and The London School of Medicine, Queen Mary University of London, London, United Kingdom; University of Miami School of Medicine, UNITED STATES

## Abstract

γδ T cells play a role in a wide range of diseases such as autoimmunity and cancer. The majority of circulating human γδ T lymphocytes express a Vγ9Vδ2+ (Vδ2+) T cell receptor (TCR) and following activation release pro-inflammatory cytokines. In this study, we show that IFNγ, produced by Vδ2+ cells, activates mesenchymal stem cell (MSC)-mediated immunosupression, which in turn exerts a negative feedback mechanism on γδ T cell function ranging from cytokine production to proliferation. Importantly, this modulatory effect is limited to a short period of time (<24 hours) post-T cell activation, after which MSCs can no longer exert their immunoregulatory capacity. Using genetically modified MSCs with the IFNγ receptor 1 constitutively silenced, we demonstrate that IFNγ is essential to this process. Activated γδ T cells induce expression of several factors by MSCs that participate in the depletion of amino acids. In particular, we show that indolamine 2,3-dioxygenase (IDO), an enzyme involved in L-tryptophan degradation, is responsible for MSC-mediated immunosuppression of Vδ2+ T cells. Thus, our data demonstrate that γδ T cell responses can be immuno-modulated by different signals derived from MSC.

## Introduction

Mesenchymal stem cells (MSCs) are multipotent non-hematopoietic precursors that can be isolated from various tissues and are capable of differentiation into multiple lineages, among them chondrocytes, adipocytes and osteocytes [1]. This notwithstanding, recent interest has focused on their potential clinical application based on their profound immunosuppressive properties. These studies have largely reported the capacity of MSCs to suppress proliferation and/or cytotoxic effector functions of distinct cells types of the innate and adaptive immune systems, such as T cells, Natural Killer (NK) cells, B cells and dendritic cells [2–8]. These properties are already being tested in numerous clinical trials worldwide. So far, none have reported significant side effects related to the transplantation of MSCs, which has encouraged the initiation of trials to treat practically any disease with links to autoimmunity (e.g. graft versus host disease, pulmonary disease, solid organ transplant, rheumatoid arthritis or systemic lupus erythematosus) [5, 8–11].

MSCs home specifically to injured tissues, attracted by pro-inflammatory cytokines [3, 12]. The immunosuppressive capacity of MSCs is not constitutive, but rather induced by crosstalk with cells of the immune system; thus, the inflammatory environment, and in particular the immune cells involved in each phase of an immune response, are likely to be critical triggers of this regulatory process. In recent years, several reports have demonstrated the role of interleukin-1 (IL-1), IFNγ and TNFα as main factors in this process [5, 13–16]. Thus, it is likely that induction of immunosuppression is not dependent on a single factor, but instead results from multiple regulatory mechanisms without an obvious hierarchy of importance. These molecules are clearly able to activate molecular pathways that increase production of soluble immunomodulatory factors such as indoleamine 2,3-deoxigenase (IDO) [3, 17], prostaglandin E2 [18], iNOS (the murine counterpart of IDO) [13], transforming growth factor β (TGFβ), hepatocyte growth factor [4], human lymphocyte Ag molecule 5, and IL-10 [19]. The influence of these MSC-secreted factors on the immune system has been recently reviewed [20].

Regarding the targets of MSC-mediated immunoregulation, most work in the field has focused on conventional T cells (αβ T cells). By contrast, the effects of MSCs on γδ T cells have not been elucidated. γδ T cells express both the γδ TCR and natural killer receptors (e.g. NKG2D), and represent a link between innate and adaptive immunity [21, 22]. In humans, γδ T cells are usually sub-divided based on use of one of two variable regions of the TCRδ-chain; Vδ1+ γδ T cells are largely found in epithelial layers such as skin and intestine, while Vδ2+ γδ T cells are mainly present in peripheral blood [23]. Most circulating Vδ2+ cells also use a Vγ9-containing TCRγ-chain, and are potently activated by low molecular weight non-peptidic phosphoantigens such a (E)-4-hydroxy-3-methyl-but-2-enyl pyrophosphate (HMBPP), an intermediate metabolite from microbial isoprenoid biosynthesis. Vδ2+ cells have the ability to produce a variety of cytokines that regulate inflammation, eliminate pathogens, and maintain tissue homeostasis [21, 24]. However, despite their beneficial roles, they have been implicated, like their αβ T cell counterparts, in the pathogenesis of a number of inflammatory diseases such as lupus erythematosus, rheumatoid arthritis, and psoriasis [25–29].

Several reports have demonstrated the inhibitory function of human bone marrow MSCs on Vδ2+ cells, mainly through PGE2 [30–34]. All of these studies used chemical inhibitors to identify and discriminate between different effector molecules secreted by MSCs. Since activated Vδ2+ cells produce pro-inflammatory cytokines upon activation, we aimed to elucidate to what degree other pathways were involved in MSC-mediated immunoregulation. Here, we report that the IFNγ/IDO pathway is a key factor for MSC-induced immunoregulation of Vδ2+ cells.

## Materials and Methods

### MSC culture

Human bone marrow-derived MSCs were obtained from the Inbiobank Stem Cell Bank (http://www.inbiomed.org/Index.php/servicios_externos/inbiobank) as described previously [35]. In short, cadaveric marrow was obtained from brain-dead donors after informed consent and under the Spanish National Organization of Transplant supervision (ONT). MSCs were positive for CD29, CD73, CD90, CD105, CD166 and CD146 but negative for markers of the hematopoietic lineage; CD34, CD45, CD14, CD19 and CD31. Moreover, they displayed a fibroblast-like phenotype and showed at least a tri-lineage potential differentiating into osteocytes, chondrocytes and adipocytes. MSCs were cultured in DMEM low-glucose medium supplemented with 10% FBS (Lonza, Walkersville, MD, USA), 2 mM glutamine, 100 U/ml penicillin and 0.1 mg/ml streptomycin (Sigma, St. Louis, MO, USA). Upon reaching confluence MSCs were treated with 0.25% Trypsin-EDTA solution (Sigma) and seeded at a density of 1000–1500 MSC/cm^2^. Cells were obtained from the Inbiobank Stem Cell Bank at passage three and all experiments were carried out with cells from low passages (passage number 4–8).

### PBMC

Peripheral blood from healthy donors was obtained after informed consent from the Basque Biobank for Research OEHUN (http://www.biobancovasco.org). Mononuclear cells (MNCs) were prepared using Ficoll Paque (Lymphprep, Axis-Shield, Oslo, Norway) according to the manufacturer’s instructions. Cells were cultured in RPMI 1640 Dutch modification (Gibco, Grand Island, NY, USA) supplemented with 10% FBS (Lonza), 2 mM glutamine, 100 U/ml penicillin, 0.1 mg/ml streptomycin (Sigma), 10 ng/ml recombinant human Interleukin-2 (IL-2, R&D, Minneapolis, MN, USA), and 1 mM (E)-4-hydroxy-dimethylallyl pyrophosphate (HDMAPP, Cayman Chemical) for the indicated times. In co-culture MNCs were activated and cultured in the presence of MSCs for the indicated times and ratios under the conditions described above. Co-culture was performed either in direct cell-to-cell contact or MNC were separated from MSCs by a transwell system (Corning, NY, USA). The inhibitor 1-Methyl-DL-tryptophan (1mM) (Sigma) was added at initiation of the co-culture.

### Lentiviral transduction of MSCs

Oligonucleotide sequences were validated at the RNAi Consortium and were purchased from Sigma. Primer sequences were as follows: IFNγRi: Fwd 5’- CATGAACCCTATCGTATATTG and Rev 5’- CATGAACCCTATCGTATATTG; IDOi: Fwd 5’- ACTGGAACTGCCTCCTATT and Rev 5’- AATGGAACTGCCTCCTATT. After annealing, the respective primer pairs were first cloned into the pSUPER plasmid and subsequently sub-cloned into the pLVTHM vector (Addgene, Cambridge, MA, USA). Viral particles were produced using the Viral Vector Platform at Inbiomed Foundation (http://www.inbiomed.org) and MSCs were transfected at a multiplicity of infection (MOI) of 10 in order to obtain a transduction efficiency of 100%.

### Flow cytometry

Antibody targets and fluorochromes were as follows: CD4-PerCP-eFluor® 710 (clone SK3), CD8-PerCP-eFluor® 710 (clone SK1), CD3-PE-Cy7 (clone UCHT1), CD45RA-APC-eFluor® 780 (clone HI100) and CD27-APC (clone LG.7F9), all from eBioscience (San Diego, CA, USA). Vδ2 TCR-PE (clone B6) and IFNγ-FITC for intracellular staining (clone 4S.B3) were from BD Pharmingen™ (San Diego, CA, USA). Intracellular staining was done with BD Cytofix/Cytoperm™ Fixation/Permeabilization Solution Kit with BD GolgiStop™Cells (BD Cytofix/Cytoperm™ Plus). For CFSE labeling of Vδ2+ cells the Cell Trace CFSE Cell Proliferation Kit (Invitrogen, San Diego, CA, USA) was used. AnnexinV-DY634 for detection of apoptotic cell death in MSCs was from Immunostep. Surface marker expression, intracellular cytokine production, cell proliferation and AnnexinV staining were analysed on a FACSCanto (BD Biosciences, Chicago, IL, USA) using BD FACSDiva™ software for acquisition. Analysis of flow cytometry data, including CFSE tracking assays and Proliferation Index [36] were done with FlowJo software v9.5.3 (TreeStar, Ashland, OR, USA). All staining, CFSE labeling, and AnnexinV staining, were performed according to the manufacturer’s protocol.

### Real-Time quantitative PCR

Total RNA extraction from MSCs and DNAse treatment was done using the RNAqueous®-Micro Total RNA Isolation Kit (Ambion, Carlsbad, CA, USA). Reverse transcription of RNA to cDNA was performed using the High Capacity cDNA Reverse Transcription Kit (Applied Biosystems, Carlsbad, CA, USA) following the manufacturer’s instructions. Quantitative Real-Time PCR was carried out on a Thermocycler Step One Plus (Applied Biosystems) using 5x PyroTaq PROBE qPCR Mix Plus (ROX) from CMB. Data were normalized to MSC-PLVTHM using the ΔΔCt method and GAPDH as housekeeping gene. Primer sequences were as follows: IFNR Fwd 5’- TCCAGGCATGCATACCGAAGACAA and Rev 5’- ATGCTGCCAGGTTCAGACTGGTTA; IDO Fwd 5’-CTACCATCTGCAAATCGTGACTAAGT and Rev 5’- GAAGGGTCTTCAGAGGTCTTATTCTC; GAPDH Fwd 5’–TGCACCACCAACTGCTTAGC and Rev 5’- GGCATGGACTGTGGTCATGAG.

### Statistical analysis

Data were summarized by mean and standard deviation. Statistical analyses were conducted using the paired t-test. p-values less than 0.05 were considered statistically significant.

## Results

### MSCs inhibit expansion of Vδ2+ cells mainly by soluble mediators

**Fig 1:**
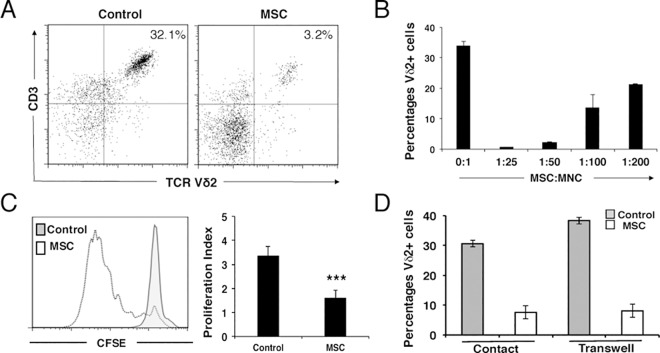
MSCs inhibit the expansion of Vδ2+ cells by soluble mediators. (A) The presence of MSCs reduces the expansion of Vδ2+ cells. Representative flow cytometric analysis of Vδ2+ cells activated from whole PBMC by HDMAPP and rh-IL2 for seven days in the absence (left panel) or presence (right panel) of MSCs. (B) Increasing ratios of MSC:MNC diminish the inhibitory effects of MSCs on Vδ2+ cell proliferation. Results show the means ± S.D. of triplicate samples. (C) Total PBMCs were labeled with CFSE and activated by HDMAPP and rh-IL2. Analysis of Vδ2+ cell proliferation in the presence (white) or absence (grey) of MSCs was performed after five days by Flow Cytometry. The presence of MSCs lowers the proliferation index of Vδ2+ cells (right panel). Results show the means ± S.D. of triplicate samples. ***P ≤ 0.001. (D) Analysis of the percentage of Vδ2+ cells cultured in cell-to-cell contact or in a transwell system in the presence/absence of MSCs. Vδ2+ cell expansion is inhibited in the same way in both systems indicating that soluble factors are responsible for immunoregulation. Results show the means ± S.D. of triplicate samples.

Vγ9Vδ2+ (Vδ2+) cells were activated from total PBMCs using HDMAPP and rh-IL2, and co-cultured with increasing numbers of MSCs. After 7-days the mononuclear fraction enriched in Vδ2+ cells was collected and analysed by flow cytometry; gating out CD4+ and CD8+ T cells, and focusing on CD3+Vδ2+ cells. As shown in Fig 1A and 1B, MSCs inhibited the expansion of Vδ2+ cells in a dose-dependent manner. As an MSC:MNC ratio of 1:25 completely abolished Vδ2+ cell proliferation, we decided to use this condition thereafter. As expected, CFSE-labeled activated Vδ2+ cells were unable to expand in the presence of MSCs, in contrast to activated Vδ2+ cells cultured without MSCs (Fig 1C), suggesting that this was not related to cell death. Proliferation of Vδ2+ cells without MSCs gave a proliferation index of 3.35 ± 0.39 as shown by decline of CFSE fluorescence. By contrast, activation of Vδ2+ cells in the presence of MSCs led to a proliferation index three times lower than control (1.59 ± 0.34) (Fig 1C, right panel). Next, we determined whether soluble factors secreted by activated Vδ2+ cells or cell-to-cell contact were responsible for the observed effect. Activated Vδ2+ cells were co-cultured with MSCs either in direct contact or in transwell dishes. As shown in Fig 1E, MSCs inhibited the expansion of Vδ2+ cells in both types of culture demonstrating that soluble molecules were responsible for the induction of immunoregulation.

### IFNγ signalling is necessary for induction of immunosuppression by MSC

**Fig 2:**
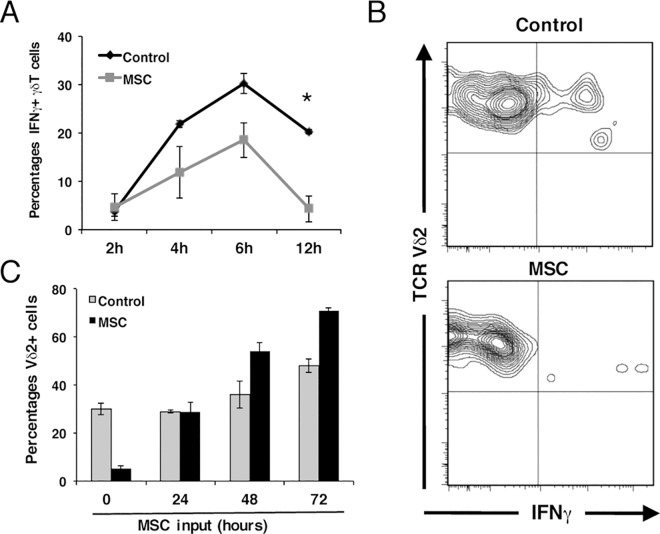
Immunoregulation is subject to a critical time window and IFNγ signalling in MSC is required for the inhibition of Vδ2+ proliferation. (A) Analysis of intracellular IFNγ production in activated Vδ2+ cells at early time points. Results show the means ± S.D. of triplicate samples. *P ≤ 0.05. (B) Representative analysis of intracellular IFNγ production by Vδ2+ cells in the absence (upper panel) or presence (lower panel) of MSCs after 12h of co-culture. (C) Inhibition of proliferation of Vδ2+ cells by MSCs in a critical time window. Whole PBMCs were pre-activated for the indicated times and subsequently co-cultured with MSCs. Percentage of Vδ2+ cells were determined by Flow Cytometry five days after plating. Results show the means ± S.D. of triplicate samples.

The induction of immunosuppression by MSCs on Vδ2+ cells can be attributed to various factors. As activation *in vitro* of Vδ2+ cells rapidly induces a production of pro-inflammatory cytokines such as IFNγ [37–40], we decided to investigate the role of this cytokine in the induction of MSC-mediated immunomodulation. As shown in Fig 2A and 2B, MSCs caused a significant reduction in IFNγ production by activated Vδ2+ cells even after short periods of time (4, 6 and 12 hours). Interestingly, when Vδ2+ cells were pre-activated for shorter than 12 hours (data not shown) or longer than 24 hours, MSCs failed to show any inhibitory effect (Fig 2C). Indeed, pre-activation with HDMAPP and IL-2 for longer than 48 hours gave rise to an opposite effect when in the presence of MSCs, as higher percentages of Vδ2+ cells were observed, probably due to production of pro-survival factors by the MSCs (e.g. IL-6) [41]. These data indicate that MSCs exert their inhibitory effect during a critical time window after which they cannot develop their regulatory capacity.

**Fig 3:**
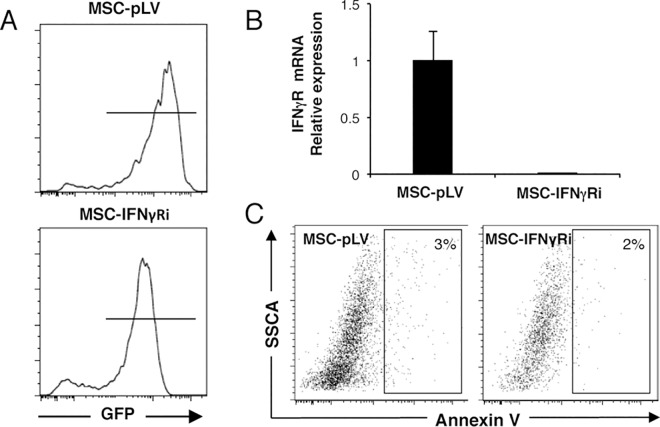
Interfering with the IFNγ pathway in MSCs by specific silencing of the IFNγR and analysis of apoptosis in MSCs. (A) Lentiviral-mediated shRNA transduction of MSCs was used to knock down the IFNγR. MSCs were transduced either with the empty vector (MSC-pLV) (upper panel) as control or with an IFNγR specific shRNA (MSC-IFNγRi) (lower panel). GFP expression in transduced cells was analysed four days after transduction by Flow Cytometry. (B) The efficiency of gene silencing was quantified by Real-Time qPCR in MSCs transduced either with the empty vector or with the one specific for IFNγRi. mRNA expression for IFNγR was significantly reduced in MSC-IFNγRi compared to MSC-pLV. Relative expression was normalized to the empty vector (MSC-pLV). (C) Transduction of MSCs does not lead to apoptotic cell death. Flow cytometric analysis of apoptotic cells (APC positive cells) in MSC-pLV (left) and MSC-IFNγRi (right) by AnnexinV staining 6 days after transduction.

**Fig 4:**
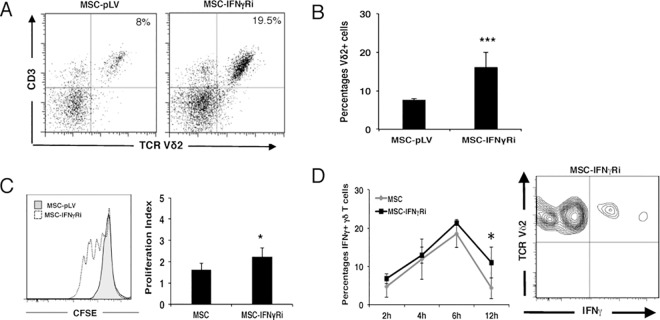
Interfering with the IFNγ pathway in MSCs can partially restore Vδ2+ cell proliferation (A) Comparison of Vδ2+ cell expansion in co-culture with either MSC-pLV (left panel) or MSC-IFNγRi (right panel). Representative Flow cytometric analysis of Vδ2+ cells at day five. (B) Specific silencing of the IFNγR augments the number of Vδ2+ cells after five days of co-culture. Results show the means ± S.D. of triplicate samples. ***P ≤ 0.001. (C) Total PBMCs were labeled with CFSE and flow cytometric analysis of Vδ2+ cell proliferation was performed five days after co-culture with either MSC-IFNγRi (white) or MSC-pLV (grey). Proliferation index of Vδ2+ cells in co-culture with MSC silenced with shRNA for IFNγR is higher compared to MSC transduced with the empty vector (right panel). Results show the means ± S.D. of triplicate samples. *P ≤ 0.05. (D) Co-culture of Vδ2+ cells and MSC-IFNγRi gives rise to more intracellular IFNγ production by Vδ2+ cells compared to a co-culture with MSC-pLV, especially after 12h. Results show the means ± S.D. of triplicate samples. *P ≤ 0.05. Representative flow cytometric analysis of IFNγ production in Vδ2+ cells after 12h of activation (right panel).

We next analysed whether inhibition of IFNγ receptor expression correlated with decreased capacity of MSCs to regulate Vδ2+ cell proliferation. We used a GFP-expressing lentiviral vector (pLVTHM) to transduce MSCs with a shRNA that targeted the IFNγ receptor. Fig 3A shows a representative FACS plot of GFP expression in MSCs 4 days after shRNA transduction. A multiplicity of infection (MOI) of 10 resulted in ~90% of cells being transduced with either empty vector (MSC-pLV) or pLVTHM-IFNγR (MSC-IFNγRi). Stable transduction resulted in a significant reduction (to less than 5% of control) of IFNγ receptor expression in MSCs (Fig 3B). Transduction did not lead to increased cell death as shown by AnnexinV staining (Fig 3C). More importantly, Vδ2+ cells expanded by ~2-fold more in co-cultures with MSCs transduced with shRNA against the IFNγ receptor, compared to co-cultures with MSCs transduced by empty vector MSC-pLV (Fig 4A and 4B). This result also correlated with data obtained by CFSE staining (Fig 4C); the proliferation index of Vδ2+ cells being increased 2-fold when the IFNγ receptor was silenced in MSCs (2.23 ±0.43 in MSC-IFNγRi vs 1.59 ±0.34 in MSC-pLV). Interestingly, cytokine production by Vδ2+ cells was partially restored by inhibition of IFNγ receptor expression in MSC, demonstrating statistical significance when we compared MSC-pVL vs. MSC-IFNγRi at 12 hours post-activation (Fig 4D). Taken together, our data demonstrate a feedback loop in which IFNγ produced by activated Vδ2+ cells can induce immunosuppressive capacity in MSCs, which in turn, can inhibit both proliferation and cytokine production of activated Vδ2+ cells.

### IDO expression by MSC induces immunosuppression of Vδ2+ cells

**Fig 5:**
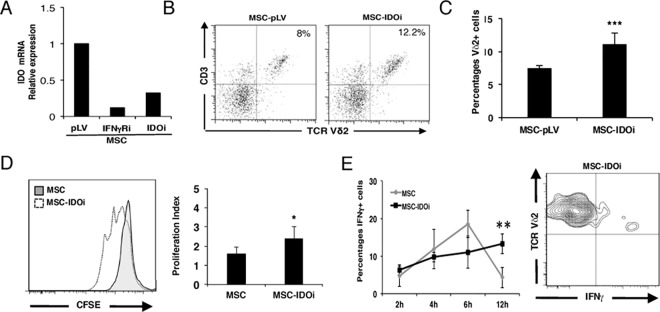
IFNγ-induced expression of IDO by MSCs is necessary to inhibit Vδ2+ cell proliferation. (A) Lentiviral-mediated shRNA transduction of MSCs was used to knock down IDO (MSC-IDOi). Quantification of IDO mRNA by Real-Time qPCR was done in MSC-pLV, MSC-IFNγRi and MSC-IDOi. Relative expression was normalized to MSC-transduced with the empty vector (B) Representative flow cytometric analysis of activated Vδ2+ cells after five days of co-culture with transduced MSCs. Vδ2+ cells expand more in the presence of MSC-IDOi compared to MSC-pLV. (C) Specific silencing of IDO augments the number of Vδ2+ cells after five days of co-culture. Results show the means ± S.D. of triplicate samples. **P ≤ 0.01. (D) Representative proliferation analysis of CFSE-labeled Vδ2+ cells performed at day five of co-culture with either MSC-IDOi (white) or empty vector (grey). Proliferation index of Vδ2+ cells in co-culture with MSCs silenced with shRNA for IDO is higher compared to MSC transduced with the empty vector (right panel), similar to the results obtained with MSC-IFNγRi. Results show the means ± S.D. of triplicate samples. *P ≤ 0.05. (E) Co-culture of Vδ2+ cells and MSC-IDOi gives rise to more intracellular IFNγ production of Vδ2+ cells compared to a co-culture with MSC-pLV after 12h. Results show the means ± S.D. of triplicate samples. **P ≤ 0.01. Representative flow cytometric analysis of IFNγ production in Vδ2+ cells co-cultured with MSC-IDOi after 12h of activation (right panel).

To further investigate the importance of IFNγ for induction of MSC-mediated immunosuppression we modulated signalling downstream of its receptor. The expression of indoleamine 2,3-deoxigenase (IDO), an enzyme involved in tryptophan catabolism, is able to modulate αβ T cell activity in response to IFNγ [3, 5, 17]. Thus, we asked whether IDO is also a key player in the immunoregulation of Vδ2+ cells by MSCs by applying the same experimental approach as described above; IDO expression was silenced using shRNA delivered by a lentiviral vector. As IDO is not constitutively expressed in MSC [5, 42], transcription was assessed in the presence of activated Vδ2+ cells. After 24-hour co-culture, quantitative Real-Time PCR for IDO clearly showed that the expression was significantly reduced in MSC transduced with specific shRNA (MSC-IDOi) compared to cells transduced with empty vector (Fig 5A). To demonstrate that IDO expression was induced in the presence of IFNγ produced by activated Vδ2+ cells, we included “knock-down” MSCs for IFNγ receptor (MSC-IFNγRi) in the same analysis. Data shown in Fig 5A confirmed that IDO expression was also reduced in MSC-IFNγRi cells.

We next evaluated the effects that MSC-IDOi cells have on Vδ2+ cells with respect to proliferation and intracellular IFNγ production. First, we checked the expansion of Vδ2+ cells cultured in the presence MSC-IDOi. As shown in Fig 5B and 5C, interfering with the IFNγ pathway at the intracellular level augmented the percentage of Vδ2+ cells in culture. Whereas Vδ2+ cells were 5.67% ±2.44 of total cells in the presence of MSCs transduced with empty vector, they constituted 11.08% ±1.24 in the presence of MSC-IDOi (11.08%), a level comparable tot that seen in the presence of MSC-IFNγRi. Consistent with this, activated CFSE-labeled Vδ2+ cells cultured with MSC-IDOi proliferated more than those cultured with MSC-pLV (Fig 5D). Importantly, the absence of IDO enzyme in MSC-IDOi allowed Vδ2+ cells to maintain IFNγ expression levels 12-hours post-activation when compared to MSCs transduced with the empty vector (MSC-pLV) alone. Notably, silencing of IDO resulted in a slightly lower percentage of Vδ2+ cells and a lower proliferation index compared with silencing of the IFNγR, which may indicate that IFNγ production is delayed in MSC-IDOi compared to MSC-IFNγRi (Fig 5E). To summarize, our data clearly show that IFNγ via the IFNγR-IDO pathway plays an important role in the immunoregulation of Vδ2+ cells by MSCs.

## Discussion

In the past decade MSC-mediated immunoregulation of T cells has attracted increasing interest due to its potential clinical application in autoimmune pathologies. Many studies have identified molecular mechanisms that underpin the immunoregulatory properties of MSCs [43]. In this study, we focused on MSC-mediated immunoregulation on Vδ2+ γδ T cells, a diverse subset that bridges innate and adaptive immunity in terms of their activation and effector functions. We demonstrate that MSCs inhibit the proliferation of Vδ2+ cells in a dose-dependent manner. Notably, this inhibitory effect is independent of cell-to-cell contact. We did not observe an increase in Vδ2+ cells after physical separation from MSCs, indicating that soluble mediators likely drive the immunoregulatory effects. Likewise, the immunomodulatory properties of MSCs are induced by cytokines secreted in their environment and/or molecules expressed by target cells such as Vδ2+ cells. Vδ2+ cells produce significant amounts of pro-inflammatory cytokines like TNFα and IFNγ in order to counteract bacterial infections or tumour development [44]. Although other studies have demonstrated MSC-mediated inhibition of both cytokines [32], we here demonstrate that IFNγ produced by Vδ2+ cells induces MSC-mediated immunosuppression of Vδ2+ cells, as judged by cytokine production and proliferation, in a negative feedback loop. Our experimental approach interfered directly with the IFNγ pathway in MSCs by silencing IFNγR1, one of the subunit of the IFNγR involved in IFNγ signalling. We showed that in the presence of MSC that lacked IFNγR1, we partially restored Vδ2+ cell expansion and intracellular IFNγ production, in contrast to the when MSC were transduced with empty vector (MSC-pLV) alone. These results indicate that the inhibitory effect exerted by MSCs on Vδ2+ cells can affect different aspects of activation and effector function, ranging from cytokine production to proliferation. Interestingly, this mechanism does not appear to be specific for Vδ2+ cells; other studies have reported that MSCs inhibit other cells (such as NK and dendritic cells) by limiting early production of IFNγ and TNFα [18, 45]. Whether this is a general anti-inflammatory mechanism employed by MSCs requires further investigation. However, we speculate that low concentrations of IFNγ are probably sufficient to promote MSC-mediated immunoregulation, which in turn, rapidly inhibits the production of this pro-inflammatory cytokine, leading to its elimination from the inflammatory environment and its capacity to active other immune cells. In this regard, it was shown that an anti-IFNγ neutralizing antibody significantly reduced the ability of HMBPP-activated Vδ2+ cells to antagonize regulatory T cell expansion [46].

Importantly, the MSC-mediated modulatory effects were limited to a short time window (<24 hours), after which MSCs appear to have no effect on the expansion of Vδ2+ cells. It is noteworthy that we have observed the same effect on activated αβ T cells (data not shown). This correlates with the antigen-presenting properties of MSCs that also occurs during a narrow window at low levels of IFN-γ [47]. Further investigations are required to determine if these observations *in vitro* are related, and how they translate to disease settings *in vivo*.

IFNγ can, through IFNγR, activate several genes that contain interferon-response-elements (IRE) in their promoter regions [48, 49]. Among these is IDO, which catalyzes the degradation of L-tryptophan to N-formylkynurenine, and has been reported to suppress αβ T cell responses [50–52]. In our hands, IFNγR and IDO gene knock-down in MSCs gave similar results. These data contrast those obtained by Martinet *et al*., that showed that a specific chemical inhibitor of IDO (1-methyl-DL-tryptophan (1-MT)) had no effect on MSC-mediated inhibition of Vδ2+ cells [31]. This may be due to differences in experimental design; one explanation could be that different phosphoantigens have been used for Vδ2+ cell activation (BrHPP vs. HDMPP in our study). Second and maybe more importantly, we have observed that 1-MT by itself has a profound effect on proliferation of Vδ2+ cells (S1 Fig). However, it is clear from both studies that several mechanisms are operating in Vδ2+ cells immunosuppression: activated Vδ2+ cells produce IFNγ and TNFα, which are able to induce IDO (mainly by IFNγ) and COX2 (IFNγ and TNFα [31]) in MSCs.

Most studies that aim to elucidate the molecules responsible for MSC-mediated immunoregulation have been performed on αβ T cells. Similar to αβ T cells, human Vδ2+ cells display remarkable functional plasticity, with reports describing production of a range of effector molecules that depends on the conditions of activation [23]. How MSCs effect this functional differentiation *in vivo*, and the implications for health and disease, are still unknown. γδ T cells were previously implicated in pathogenesis in animal models of autoimmune diseases such as rheumatoid arthritis and multiple sclerosis in which Th1 cytokines are thought to play a central role [53, 54]. Moreover, they contribute to other autoimmune diseases such as psoriasis in which epidermal CCR6+ Vδ2+ cells express high levels of IL-17 and IL-22 [55]. IL-17+ Vδ2+ cells are known to express low levels of IFNγ [56]. Hence, it remains to be determined how these different subtypes of Vδ2+ cells would interact with MSCs, which molecules would be involved, and to what extent the role of MSCs might be beneficial in the immunotherapy of Vδ2+ cell-mediated diseases.

Finally, our findings also contrast previous reports suggesting that MSC-associated immunosuppression of Vδ2+ cells is exclusively mediated by PGE2. As shown in this study, IDO is also involved, suggesting that several pathways underpin the immunomodulatory capacities of MSCs.

## Supporting Information

S1 Fig Total PBMCs were activated and cultured in the presence or absence of MSCs. Addition of 1-MT alone reduces significantly the expansion of Vδ2+ cells even in the absence of MSCs while addition of vehicle has no influence on the percentage of Vδ2+ cells. Results show the means ± S.D. of triplicate samples.
(TIF)

## Abbreviations

MSCs: mesenchymal stem cells
TNFα: tumor necrosis factor alpha
IFNγ: Interferon gamma
IFNR: IFNγ receptor
TCR: T cell receptor

## References

[1] Pittenger MF, Mackay AM, Beck SC, Jaiswal RK, Douglas R, Mosca JD, et al Multilineage potential of adult human mesenchymal stem cells. Science. 1999;284(5411):143–7. Epub 1999/04/02. 10102814 10.1126/science.284.5411.143

[2] Corcione A, Benvenuto F, Ferretti E, Giunti D, Cappiello V, Cazzanti F, et al Human mesenchymal stem cells modulate B-cell functions. Blood. 2006;107(1):367–72. Epub 2005/09/06. 10.1182/blood-2005-07-2657 16141348

[3] DelaRosa O, Lombardo E, Beraza A, Mancheno-Corvo P, Ramirez C, Menta R, et al Requirement of IFN-gamma-mediated indoleamine 2,3-dioxygenase expression in the modulation of lymphocyte proliferation by human adipose-derived stem cells. Tissue Eng Part A. 2009;15(10):2795–806. Epub 2009/02/24. 10.1089/ten.TEA.2008.0630 19231921

[4] Di Nicola M, Carlo-Stella C, Magni M, Milanesi M, Longoni PD, Matteucci P, et al Human bone marrow stromal cells suppress T-lymphocyte proliferation induced by cellular or nonspecific mitogenic stimuli. Blood. 2002;99(10):3838–43. Epub 2002/05/03. 11986244 10.1182/blood.v99.10.3838

[5] Dorronsoro A, Fernandez-Rueda J, Fechter K, Ferrin I, Salcedo JM, Jakobsson E, et al Human Mesenchymal Stromal Cell-Mediated Immunoregulation: Mechanisms of Action and Clinical Applications. Bone marrow research. 2013;2013:203643 PubMed Central PMCID: PMC3804286. 10.1155/2013/203643 24187625 PMC3804286

[6] Nauta AJ, Kruisselbrink AB, Lurvink E, Willemze R, Fibbe WE. Mesenchymal stem cells inhibit generation and function of both CD34+-derived and monocyte-derived dendritic cells. Journal of immunology. 2006;177(4):2080–7. Epub 2006/08/05. 10.4049/jimmunol.177.4.2080 16887966

[7] Sotiropoulou PA, Perez SA, Gritzapis AD, Baxevanis CN, Papamichail M. Interactions between human mesenchymal stem cells and natural killer cells. Stem Cells. 2006;24(1):74–85. Epub 2005/08/16. 10.1634/stemcells.2004-0359 16099998

[8] Krampera M. Mesenchymal stromal cells: more than inhibitory cells. Leukemia: official journal of the Leukemia Society of America, Leukemia Research Fund, UK. 2011;25(4):565–6. Epub 2011/04/14. 10.1038/leu.2011.8 21487446

[9] Antunes MA, Laffey JG, Pelosi P, Rocco PR. Mesenchymal Stem Cell Trials for Pulmonary Diseases. Journal of cellular biochemistry. 2014. 10.1002/jcb.24783 24515922

[10] Keating A. Mesenchymal stromal cells: new directions. Cell stem cell. 2012;10(6):709–16. Epub 2012/06/19. 10.1016/j.stem.2012.05.015 22704511

[11] Le Blanc K, Mougiakakos D. Multipotent mesenchymal stromal cells and the innate immune system. Nature reviews Immunology. 2012;12(5):383–96. 10.1038/nri3209 22531326

[12] Carrero R, Cerrada I, Lledo E, Dopazo J, Garcia-Garcia F, Rubio MP, et al IL1beta induces mesenchymal stem cells migration and leucocyte chemotaxis through NF-kappaB. Stem cell reviews. 2012;8(3):905–16. Epub 2012/04/03. PubMed Central PMCID: PMC3412085. 10.1007/s12015-012-9364-9 22467443 PMC3412085

[13] Carcamo-Orive I, Tejados N, Delgado J, Gaztelumendi A, Otaegui D, Lang V, et al ERK2 protein regulates the proliferation of human mesenchymal stem cells without affecting their mobilization and differentiation potential. Experimental cell research. 2008;314(8):1777–88. Epub 2008/04/02. 10.1016/j.yexcr.2008.01.020 18378228

[14] Krampera M, Cosmi L, Angeli R, Pasini A, Liotta F, Andreini A, et al Role for interferon-gamma in the immunomodulatory activity of human bone marrow mesenchymal stem cells. Stem Cells. 2006;24(2):386–98. Epub 2005/08/27. 10.1634/stemcells.2005-0008 16123384

[15] Groh ME, Maitra B, Szekely E, Koc ON. Human mesenchymal stem cells require monocyte-mediated activation to suppress alloreactive T cells. Experimental hematology. 2005;33(8):928–34 10.1016/j.exphem.2005.05.002 16038786

[16] English K, Barry FP, Field-Corbett CP, Mahon BP. IFN-gamma and TNF-alpha differentially regulate immunomodulation by murine mesenchymal stem cells. Immunol Lett. 2007;110(2):91–100. Epub 2007/05/18. 10.1016/j.imlet.2007.04.001 17507101

[17] Meisel R, Zibert A, Laryea M, Gobel U, Daubener W, Dilloo D. Human bone marrow stromal cells inhibit allogeneic T-cell responses by indoleamine 2,3-dioxygenase-mediated tryptophan degradation. Blood. 2004;103(12):4619–21. Epub 2004/03/06. 10.1182/blood-2003-11-3909 15001472

[18] Aggarwal S, Pittenger MF. Human mesenchymal stem cells modulate allogeneic immune cell responses. Blood. 2005;105(4):1815–22. 10.1182/blood-2004-04-1559 15494428

[19] Yang SH, Park MJ, Yoon IH, Kim SY, Hong SH, Shin JY, et al Soluble mediators from mesenchymal stem cells suppress T cell proliferation by inducing IL-10. Exp Mol Med. 2009;41(5):315–24. Epub 2009/03/25. 10.3858/emm.2009.41.5.035 19307751 PMC2701980

[20] Soleymaninejadian E, Pramanik K, Samadian E. Immunomodulatory properties of mesenchymal stem cells: cytokines and factors. American journal of reproductive immunology. 2012;67(1):1–8. 10.1111/j.1600-0897.2011.01069.x 21951555

[21] Tanaka Y, Morita CT, Tanaka Y, Nieves E, Brenner MB, Bloom BR. Natural and synthetic non-peptide antigens recognized by human gamma delta T cells. Nature. 1995;375(6527):155–8. Epub 1995/05/11. 10.1038/375155a0 7753173

[22] Tanaka Y, Sano S, Nieves E, De Libero G, Rosa D, Modlin RL, et al Nonpeptide ligands for human gamma delta T cells. Proceedings of the National Academy of Sciences of the United States of America. 1994;91(17):8175–9. PubMed Central PMCID: PMC44568. 8058775 10.1073/pnas.91.17.8175 PMC44568

[23] Pang DJ, Neves JF, Sumaria N, Pennington DJ. Understanding the complexity of gammadelta T-cell subsets in mouse and human. Immunology. 2012;136(3):283–90. Epub 2012/03/06. PubMed Central PMCID: PMC3385028. 10.1111/j.1365-2567.2012.03582.x 22385416 PMC3385028

[24] Shen Y, Zhou D, Qiu L, Lai X, Simon M, Shen L, et al Adaptive immune response of Vgamma2Vdelta2+ T cells during mycobacterial infections. Science. 2002;295(5563):2255–8. Epub 2002/03/23. PubMed Central PMCID: PMC2872146. 10.1126/science.1068819 11910108 PMC2872146

[25] Cai Y, Shen X, Ding C, Qi C, Li K, Li X, et al Pivotal role of dermal IL-17-producing gammadelta T cells in skin inflammation. Immunity. 2011;35(4):596–610. PubMed Central PMCID: PMC3205267. 10.1016/j.immuni.2011.08.001 21982596 PMC3205267

[26] Hu C, Qian L, Miao Y, Huang Q, Miao P, Wang P, et al Antigen-presenting effects of effector memory Vgamma9Vdelta2 T cells in rheumatoid arthritis. Cellular & molecular immunology. 2012;9(3):245–54. 22139198 10.1038/cmi.2011.50 PMC4012843

[27] Laggner U, Di Meglio P, Perera GK, Hundhausen C, Lacy KE, Ali N, et al Identification of a novel proinflammatory human skin-homing Vgamma9Vdelta2 T cell subset with a potential role in psoriasis. Journal of immunology. 2011;187(5):2783–93. PubMed Central PMCID: PMC3187621. 10.4049/jimmunol.1100804 PMC3187621 21813772

[28] Su D, Shen M, Li X, Sun L. Roles of gammadelta T cells in the pathogenesis of autoimmune diseases. Clinical & developmental immunology. 2013;2013:985753. PubMed Central PMCID: PMC3600234. 23533458 10.1155/2013/985753 PMC3600234

[29] Sutton CE, Lalor SJ, Sweeney CM, Brereton CF, Lavelle EC, Mills KH. Interleukin-1 and IL-23 induce innate IL-17 production from gammadelta T cells, amplifying Th17 responses and autoimmunity. Immunity. 2009;31(2):331–41. 10.1016/j.immuni.2009.08.001 19682929

[30] Liu X, Feng T, Gong T, Shen C, Zhu T, Wu Q, et al Human Umbilical Cord Mesenchymal Stem Cells Inhibit the Function of Allogeneic Activated Vgamma9Vdelta2 T Lymphocytes In Vitro. BioMed research international. 2015;2015:317801 PubMed Central PMCID: PMC4423519. 10.1155/2015/317801 25984529 PMC4423519

[31] Martinet L, Fleury-Cappellesso S, Gadelorge M, Dietrich G, Bourin P, Fournie JJ, et al A regulatory cross-talk between Vgamma9Vdelta2 T lymphocytes and mesenchymal stem cells. European journal of immunology. 2009;39(3):752–62. Epub 2009/02/07. 10.1002/eji.200838812 19197941

[32] Martinet L, Jean C, Dietrich G, Fournie JJ, Poupot R. PGE2 inhibits natural killer and gamma delta T cell cytotoxicity triggered by NKR and TCR through a cAMP-mediated PKA type I-dependent signaling. Biochem Pharmacol. 2010;80(6):838–45. Epub 2010/05/18. 10.1016/j.bcp.2010.05.002 20470757

[33] Petrini I, Pacini S, Petrini M, Fazzi R, Trombi L, Galimberti S. Mesenchymal cells inhibit expansion but not cytotoxicity exerted by gamma-delta T cells. Eur J Clin Invest. 2009;39(9):813–8. Epub 2009/06/16. 10.1111/j.1365-2362.2009.02171.x 19522834

[34] Prigione I, Benvenuto F, Bocca P, Battistini L, Uccelli A, Pistoia V. Reciprocal interactions between human mesenchymal stem cells and gammadelta T cells or invariant natural killer T cells. Stem Cells. 2009;27(3):693–702. Epub 2008/12/20. 10.1634/stemcells.2008-0687 19096038

[35] Carcamo-Orive I, Gaztelumendi A, Delgado J, Tejados N, Dorronsoro A, Fernandez-Rueda J, et al Regulation of human bone marrow stromal cell proliferation and differentiation capacity by glucocorticoid receptor and AP-1 crosstalk. Journal of bone and mineral research: the official journal of the American Society for Bone and Mineral Research. 2010;25(10):2115–25. Epub 2010/05/26. PubMed Central PMCID: PMC3607410. 10.1002/jbmr.120 PMC3607410 20499359

[36] Roederer M. Interpretation of cellular proliferation data: avoid the panglossian. Cytometry Part A: the journal of the International Society for Analytical Cytology. 2011;79(2):95–101. 21265003 10.1002/cyto.a.21010

[37] Battistini L, Borsellino G, Sawicki G, Poccia F, Salvetti M, Ristori G, et al Phenotypic and cytokine analysis of human peripheral blood gamma delta T cells expressing NK cell receptors. Journal of immunology. 1997;159(8):3723–30. 9378958

[38] Follows GA, Munk ME, Gatrill AJ, Conradt P, Kaufmann SH. Gamma interferon and interleukin 2, but not interleukin 4, are detectable in gamma/delta T-cell cultures after activation with bacteria. Infection and immunity. 1992;60(3):1229–31. PubMed Central PMCID: PMC257618. 1531813 10.1128/iai.60.3.1229-1231.1992 PMC257618

[39] Garcia VE, Sieling PA, Gong J, Barnes PF, Uyemura K, Tanaka Y, et al Single-cell cytokine analysis of gamma delta T cell responses to nonpeptide mycobacterial antigens. Journal of immunology. 1997;159(3):1328–35. 9233629

[40] Goodier MR, Lundqvist C, Hammarstrom ML, Troye-Blomberg M, Langhorne J. Cytokine profiles for human V gamma 9+ T cells stimulated by Plasmodium falciparum. Parasite immunology. 1995;17(8):413–23. 7501422 10.1111/j.1365-3024.1995.tb00909.x

[41] Xu G, Zhang Y, Zhang L, Ren G, Shi Y. The role of IL-6 in inhibition of lymphocyte apoptosis by mesenchymal stem cells. Biochemical and biophysical research communications. 2007;361(3):745–50. PubMed Central PMCID: PMC2699935. 10.1016/j.bbrc.2007.07.052 17678624 PMC2699935

[42] Ryan JM, Barry F, Murphy JM, Mahon BP. Interferon-gamma does not break, but promotes the immunosuppressive capacity of adult human mesenchymal stem cells. Clin Exp Immunol. 2007;149(2):353–63. Epub 2007/05/25. PubMed Central PMCID: PMC1941956. 10.1111/j.1365-2249.2007.03422.x 17521318 PMC1941956

[43] Duffy MM, Ritter T, Ceredig R, Griffin MD. Mesenchymal stem cell effects on T-cell effector pathways. Stem Cell Res Ther. 2011;2(4):34 Epub 2011/08/25. PubMed Central PMCID: PMC3219065. 10.1186/scrt75 21861858 PMC3219065

[44] Chien YH, Meyer C, Bonneville M. gammadelta T Cells: First Line of Defense and Beyond. Annual review of immunology. 2014. 10.1146/annurev-immunol-032713-120216 24387714

[45] Spaggiari GM, Capobianco A, Abdelrazik H, Becchetti F, Mingari MC, Moretta L. Mesenchymal stem cells inhibit natural killer-cell proliferation, cytotoxicity, and cytokine production: role of indoleamine 2,3-dioxygenase and prostaglandin E2. Blood. 2008;111(3):1327–33. 10.1182/blood-2007-02-074997 17951526

[46] Gong G, Shao L, Wang Y, Chen CY, Huang D, Yao S, et al Phosphoantigen-activated V gamma 2V delta 2 T cells antagonize IL-2-induced CD4+CD25+Foxp3+ T regulatory cells in mycobacterial infection. Blood. 2009;113(4):837–45. PubMed Central PMCID: PMC2630269. 10.1182/blood-2008-06-162792 18981295 PMC2630269

[47] Chan JL, Tang KC, Patel AP, Bonilla LM, Pierobon N, Ponzio NM, et al Antigen-presenting property of mesenchymal stem cells occurs during a narrow window at low levels of interferon-gamma. Blood. 2006;107(12):4817–24. PubMed Central PMCID: PMC1895812. 10.1182/blood-2006-01-0057 16493000 PMC1895812

[48] Schroder K, Hertzog PJ, Ravasi T, Hume DA. Interferon-gamma: an overview of signals, mechanisms and functions. J Leukoc Biol. 2004;75(2):163–89. Epub 2003/10/04. 10.1189/jlb.0603252 14525967

[49] Stark GR. How cells respond to interferons revisited: from early history to current complexity. Cytokine Growth Factor Rev. 2007;18(5–6):419–23. Epub 2007/08/09. PubMed Central PMCID: PMC2081984. 10.1016/j.cytogfr.2007.06.013 17683974 PMC2081984

[50] Hwu P, Du MX, Lapointe R, Do M, Taylor MW, Young HA. Indoleamine 2,3-dioxygenase production by human dendritic cells results in the inhibition of T cell proliferation. Journal of immunology. 2000;164(7):3596–9. 10.4049/jimmunol.164.7.3596 10725715

[51] Munn DH, Shafizadeh E, Attwood JT, Bondarev I, Pashine A, Mellor AL. Inhibition of T cell proliferation by macrophage tryptophan catabolism. The Journal of experimental medicine. 1999;189(9):1363–72. PubMed Central PMCID: PMC2193062. 10224276 10.1084/jem.189.9.1363 PMC2193062

[52] Munn DH, Sharma MD, Lee JR, Jhaver KG, Johnson TS, Keskin DB, et al Potential regulatory function of human dendritic cells expressing indoleamine 2,3-dioxygenase. Science. 2002;297(5588):1867–70. 10.1126/science.1073514 12228717

[53] Rajan AJ, Gao YL, Raine CS, Brosnan CF. A pathogenic role for gamma delta T cells in relapsing-remitting experimental allergic encephalomyelitis in the SJL mouse. Journal of immunology. 1996;157(2):941–9. 8752949

[54] Spahn TW, Issazadah S, Salvin AJ, Weiner HL. Decreased severity of myelin oligodendrocyte glycoprotein peptide 33–35-induced experimental autoimmune encephalomyelitis in mice with a disrupted TCR delta chain gene. European journal of immunology. 1999;29(12):4060–71. 10.1002/(SICI)1521-4141(199912)29:12<4060::AID-IMMU4060>3.0.CO;2-S 10602017

[55] Mabuchi T, Chang TW, Quinter S, Hwang ST. Chemokine receptors in the pathogenesis and therapy of psoriasis. Journal of dermatological science. 2012;65(1):4–11. 10.1016/j.jdermsci.2011.11.007 22177422

[56] Ribot JC, deBarros A, Pang DJ, Neves JF, Peperzak V, Roberts SJ, et al CD27 is a thymic determinant of the balance between interferon-gamma- and interleukin 17-producing gammadelta T cell subsets. Nature immunology. 2009;10(4):427–36. PubMed Central PMCID: PMC4167721. 10.1038/ni.1717 19270712 PMC4167721

