# Supplementary figures and images for: IFNγ Regulates Activated Vδ2+ T Cells through a Feedback Mechanism Mediated by Mesenchymal Stem Cells
Source: PLoS One. 2017 Jan 11;12(1):e0169362. doi: 10.1371/journal.pone.0169362 (PMC5226805; doi:10.1371/journal.pone.0169362)

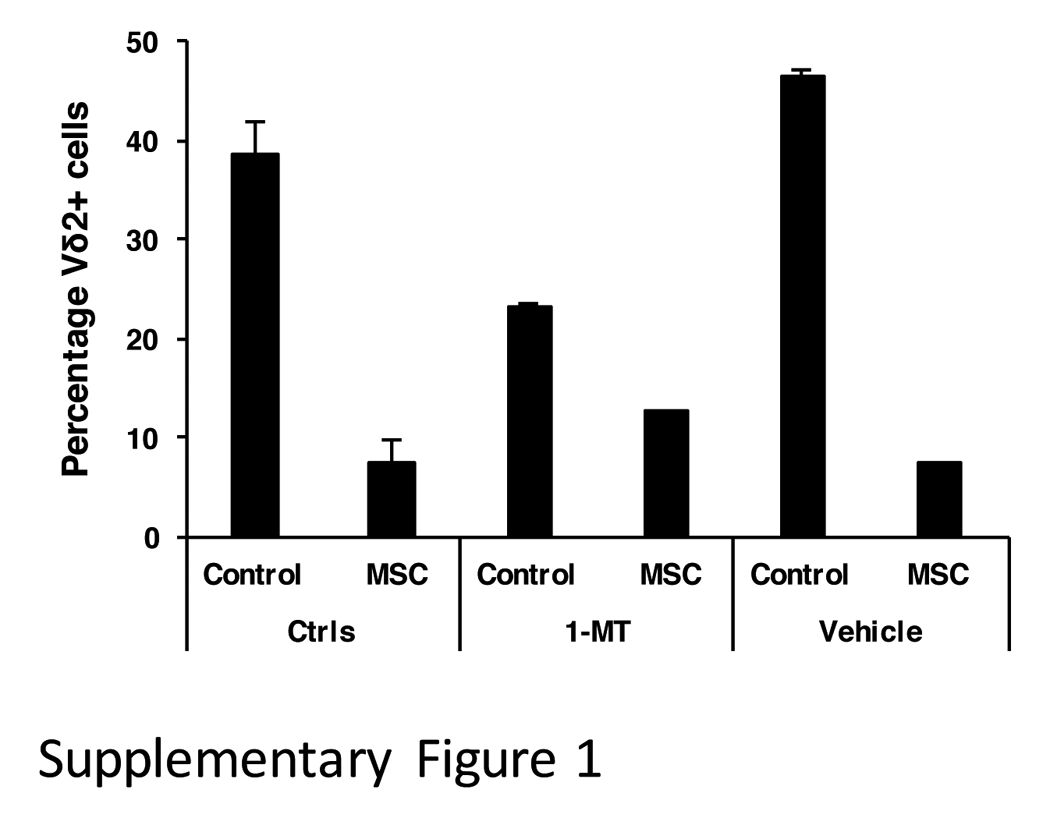

Supplement: S1 Fig — Addition of 1-MT alone reduces significantly the expansion of Vδ2+ cells even in the absence of MSCs while addition of vehicle has no influence on the percentage of Vδ2+ cells. Results show the means ± S.D. of triplicate samples. (TIF) [file pone.0169362.s001.tif]
